# Upper Airway Complications in COVID-19: A Case Series

**DOI:** 10.7759/cureus.37163

**Published:** 2023-04-05

**Authors:** Sushmita Shrestha, Yong Shin, Oleg V Sostin, Polina Pinkhasova, John Chronakos

**Affiliations:** 1 Medicine, Danbury Hospital, Danbury, USA; 2 Research and Innovation, Nuvance Health, Danbury, USA; 3 Pulmonary, Critical Care, and Sleep Medicine, Danbury Hospital, Danbury, USA

**Keywords:** laryngeal edema, tracheomalacia, tracheal stenosis, upper airway obstruction, covid-19

## Abstract

Prolonged intubation is associated with several complications leading to upper airway obstruction, including tracheal stenosis and tracheomalacia. Tracheostomy may potentially decrease the risk of tracheal injury in patients with upper airway obstruction. The ideal timing to perform tracheostomy remains controversial. Prolonged intubations were particularly common during the initial phase of the coronavirus disease 2019 (COVID-19) pandemic. This study aimed to present a series of five cases of upper airway complications in patients who underwent mechanical ventilation in the setting of COVID-19 and discuss their clinical aspects, risk factors, and therapeutic strategies.

## Introduction

Although prolonged intubations are frequently seen in patients with brainstem lesions, neuromuscular disease, and cardiopulmonary diseases, they were particularly common during the initial phase of the coronavirus disease 2019 (COVID-19) pandemic [1]. Based on the experience in China, 5% of the patients diagnosed with COVID-19 progressed to a severe disease requiring mechanical ventilation [2]. Most patients (75-88%) with severe COVID-19 infections admitted to intensive care units in Italy, Seattle, New York City, and Boston were mechanically ventilated, with a median duration of mechanical ventilation of 10 and 16 days in the Seattle and Boston cohorts, respectively [1].

Prolonged intubation is associated with several complications, including tracheal stenosis and tracheomalacia, which lead to upper airway obstruction. Tracheal stenosis may result from intrinsic compression at any anatomic level. The reported incidence of tracheal stenosis following endotracheal intubation ranges from 6% to 21% [3]. The pathophysiology is attributed to ischemic damage of the subglottic area at the level of the endotracheal tube cuff resulting in increased fibrous tissue proliferation [4]. Tracheomalacia indicates a condition characterized by weakening of the tracheal wall and excessive collapsibility of the trachea related to inflammatory processes that produce diffuse or localized tracheomalacia [5].

Tracheostomy, or the formation of an opening in the trachea allowing mechanical ventilation via a tracheostomy tube instead of an endotracheal tube, may potentially decrease the risk of tracheal injury. The ideal timing to perform tracheostomy remains controversial. Different observational studies have documented variable timing. As per a meta-analysis of randomized clinical trials, it is reasonable to wait for 10 days to ensure an ongoing need for mechanical ventilation before pursuing tracheostomy [6]. The Project IMPACT database showed a median of nine days in the intensive care unit (ICU) before pursuing the tracheostomy procedure [7]. In contrast, patients with COVID-19-associated respiratory failure routinely had tracheostomy performed after approximately 21 days of mechanical ventilation [8].

It has been our observation that COVID-19 patients on mechanical ventilation develop early and more frequent upper airway complications. In this case series, we aimed to describe the clinical characteristics of patients who required prolonged intubation in the setting of COVID-19 and developed early tracheal complications.

## Case presentation

Case 1

A 57-year-old female with a past medical history of diabetes mellitus and asthma, who tested positive for SARS-CoV-2 RNA as an outpatient, presented to the emergency department (ED) with eight days of dyspnea and fever and was admitted for acute hypoxemic respiratory failure secondary to COVID-19 pneumonia. Despite the treatment with intravenous methylprednisolone, convalescent plasma, tocilizumab, and a therapeutic dose of enoxaparin, the patient required intubation for worsening hypoxemia. She was extubated 10 days later. However, she immediately developed severe stridor requiring re-intubation, and racemic epinephrine was added to her regimen to relieve upper airway edema. The patient improved clinically and was extubated two days later and was put on 2 L/min of supplemental oxygen. Six days post-reextubation, she was found again to be in respiratory distress with inspiratory stridor requiring high-flow nasal cannula, for which she received inhaled corticosteroids and racemic epinephrine nebulization with some clinical improvement. Computed tomography (CT) of the neck showed laryngeal edema. Empiric antibiotics were added for possible bacterial laryngitis. Her laryngeal edema resolved over the next few days. The patient was discharged on systemic steroids, initiated two days prior to intubation.

Case 2

A 34-year-old female with past medical history of obesity, obstructive sleep apnea, and cognitive deficit, presented to the ED with dyspnea, tachypnea, and profound hypoxia. As her oxygenation failed to improve with high-flow oxygen supplementation and proning, she was intubated for acute respiratory failure secondary to acute respiratory distress syndrome (ARDS) in the setting of COVID-19 pneumonia, and treatment with hydroxychloroquine, ceftriaxone, azithromycin, and tocilizumab was initiated. Her clinical course was complicated by methicillin-susceptible *Staphylococcus aureus *(MSSA) pneumonia and acute kidney injury. She remained intubated for nine days, improved clinically, and was subsequently discharged home. However, two weeks later, the patient presented with respiratory distress requiring intubation due to deteriorating mental status. Her chest x-ray showed worsening airspace disease in the left lower lobe and she received convalescent plasma therapy for COVID-19. CT revealed subglottic occlusion of the tracheal lumen until just distal to the endotracheal tube (ETT). Bronchoscopy confirmed the presence of granulation tissue around the trachea. Distal ETT showed occlusion by a fragment of granulation tissue exerting a ball-valve mechanism. Friable bits of granulation tissues were suctioned, and the ETT was advanced past the stenosis with bronchoscopic guidance. The patient subsequently underwent balloon dilation and cryoablation of the subglottic larynx. She was found to have multilevel tracheal stenosis with a significant amount of exudative tracheitis. The patient improved after the procedure and was discharged home. Several days later, she presented again with dyspnea and audible inspiratory wheezing. CT of the neck revealed a 2.9 cm long segment of circumferential wall thickening in the lower trachea, with resultant severe focal tracheal stenosis. The patient failed multiple sessions of endoscopic therapy; given the significant inflammation within the airway, she was not a candidate for tracheal resection. She was treated with systemic steroids and antibiotics to reduce inflammation. Tracheal reconstruction was recommended; however, her family declined. Therefore, she underwent a tracheostomy.

Case 3

A 42-year-old male with a past medical history of Down syndrome, who tested positive for SARS-CoV-2 RNA as outpatient, presented to the ED with fever and dyspnea of seven-day duration. As his hypoxia failed to improve with high-flow oxygen supplementation, the patient was intubated for acute respiratory failure secondary to ARDS in the setting of COVID-19, and treatment with tocilizumab, intravenous dexamethasone, therapeutic dose of enoxaparin, and hydroxychloroquine was initiated. After extubation on day 12, no post-extubation stridor was noted. However, he was emergently reintubated the following day due to respiratory distress, with agonal breathing, of unclear etiology. The patient was empirically treated with antibiotics and supported by aggressive pulmonary toileting. He was successfully extubated the next day and was later discharged to home. In approximately two months, the patient returned to the hospital with dyspnea and stridor requiring re-intubation and received intravenous dexamethasone and racemic epinephrine. CT demonstrated persistent pulmonary ground-glass opacities consistent with sequelae of COVID-19 and a tracheal narrowing from the level of T1 through the superior aspect of T3 vertebrae. Bronchoscopy with balloon dilation of the tracheal segment was performed. The patient was extubated and discharged to home.

He was again readmitted nine days later with recurrent stridor and dyspnea, requiring intubation. Flexible fiberoptic bronchoscopy via the ET tube revealed a 4 cm segment of tracheomalacia that demonstrated dynamic collapse with respiration in the mid-trachea. There was a significant narrowing in the distal part of the segment, with apposition of the anterior, posterior, and lateral walls and signs of ongoing inflammation. The patient underwent placement of a 14 × 60 mm silicone tracheal stent and was extubated. He also received vancomycin, followed by daptomycin, as methicillin-resistant *S. aureus *(MRSA) was detected in the respiratory culture and he developed transient MRSA bacteremia after the stent placement. A month later, the patient underwent elective removal of the stent and tracheal balloon dilatation. However, the following day, he developed stridor requiring intubation. After three weeks, he underwent rigid laryngoscopy, using suspension and flexible bronchoscopy, showing subglottic and cervical/upper tracheal stenosis, for which he underwent temporary tracheostomy, followed by tracheoplasty two months later. However, the patient continued to have recurrent episodes of acute respiratory failure, including a respiratory arrest, attributed to mucous plugging of the ETT. Over the following month, he underwent laryngoscopy, bronchoscopy, cryoablation, and tracheal tube exchange; however, mucous plugging continued to recur. Over the following three months, repeat cryotherapy, Montgomery T-tube placement (with subsequent upsizing), dilation of the subglottic area, and balloon dilatation of the tracheal stoma were performed. However, despite these efforts, mucous plugging of the ET has remained a major problem. The patient had a pulseless cardiac arrest secondary to a distal obstruction of the T-tube due to a ball valve effect.

Case 4

A 62-year-old female with a past medical history of non-alcoholic liver cirrhosis, diabetes mellitus, and obesity presented to the ED with dyspnea and fever of seven-day duration. She was admitted with acute hypoxemic respiratory failure with high suspicion of COVID-19 pneumonia and was empirically treated with lopinavir/ritonavir and hydroxychloroquine, which were discontinued due to QT segment prolongation. As her respiratory status worsened, she was intubated and a cephalosporin was added due to concern of superimposed bacterial pneumonia. SARS-CoV-2 RNA PCR came back positive two days after the admission, and the patient received therapeutic enoxaparin, intravenous dexamethasone, and convalescent plasma. She was extubated on day 15 and re-intubated 10 days later as she developed respiratory distress and stridor non-responsive to racemic epinephrine. The intubation was difficult due to severe laryngeal edema consistent with findings on the neck CT. Extubation was attempted after a trial of inhaled steroids, but given immediately audible stridor, she was re-intubated. Tracheostomy was performed on day seven of mechanical ventilation. She subsequently developed *Escherichia coli *bacteremia of an unknown source and Pseudomonas pneumonia, complicated by right hydropneumothorax, and was started on meropenem and vancomycin; a right-sided chest tube was placed. Ultimately, the patient expired from multiorgan failure secondary to profound septic shock refractory to antibiotics and vasopressors. Autopsy revealed bilateral findings of ulcerated proximal and distal tracheal mucosa, ulcerated vocal cords, and acute pneumonia, as well as right-sided organizing pneumonia in the upper and middle lobes.

Case 5

A 32-year-old male with a past medical history of obesity and Hodgkin's lymphoma in remission presented to the ED with dyspnea, fever, and myalgias of five days, after testing positive for SARS-CoV-2 RNA as outpatient. He was found to be tachycardic and hypoxic, and CT showed diffuse ground-glass consolidations, consistent with COVID-19 pneumonia. Given progressive hypoxemia, the patient required intubation. He failed multiple extubation attempts due to persistent volume overload and ongoing COVID-19 pneumonia. Empiric antibiotic therapy was added due to the concern of superimposed bacterial pneumonia. His ICU course was further complicated by acute renal failure requiring hemodialysis. Extubation was re-attempted after volume status optimization; however, audible stridor was noted immediately after extubation. Despite a trial of steroids for presumed tracheal stenosis due to inflammation and edema, the patient remained in respiratory distress. During rapid sequence intubation, he developed pulseless ventricular fibrillation. Resuscitation, performed for over 40 minutes, was unsuccessful.

## Discussion

Many clinical reports suggest that COVID-19 patients experience extended stays in the ICU with prolonged intubations, stretching from one to two weeks or longer [9]. In the present case series, we report cases representing intubations of variable lengths. Patient characteristics are summarized in Table 1. Two patients required one week of mechanical ventilation, the other two patients required two weeks, and one patient required three weeks of ventilation.

**Table 1:** Characteristics and outcomes of patients. LRV: lopinavir/ritonavir; TOC: tocilizumab; HDC: hydroxychloroquine; MPD: methylprednisolone; DEX: dexamethasone; bid: twice a day; q: every; ARDS: acute respiratory distress syndrome; COPD: chronic obstructive pulmonary disease

Variables	Case 1	Case 2	Case 3	Case 4	Case 5
Age in years	57	34	42	62	32
Sex	Female	Female	Male	Female	Male
Weight in kg/BMI in kg/m^2^	118.5/47.3	80/35.7	34.5/24.7	93.0/39.9	140.0/43
Smoking status	Never	Never	Never	Never	Never
COPD	No	No	No	No	No
Duration of intubation, days	8, 3 (re-intubated)	9	12	15	23
Tracheal Stenosis	No	29 days post-intubation	2.5 months post-intubation	No	31 days post-intubation
Tracheomalacia	No	No	60 days post-intubation	No	No
Laryngeal edema	12 days post-intubation	No	No	61 days post-intubation	No
Tracheal procedure (days post-intubation)	None	(1) Cryoablation, balloon dilation (31, 50, 52, 79), (2) tracheostomy (87)	(1) Balloon dilation of tracheal segment (60), (2) tracheal stent placement (79), (3) tracheal balloon dilatation (120), (4) tracheostomy (146), (5) tracheoplasty (276), (6) cryoablation and tracheal tube exchange (303), (7) cryotherapy and montgomery tracheostomy (330), (8) dilation of subglottic area/tracheal stoma and replacement of tracheostomy tube (442)	Tracheostomy (30)	None
Antiviral drug	None	None	None	LRV 200 mg bid	None
Anti-inflammatory drug	TOC 800 mg once	HDC 200 mg bid for 5 days; TOC 800 mg once	HDC 200 mg bid for 5 days; TOC 800 mg once	HDC 200 mg for 2 days	HDC 200 mg for 5 days
Systemic steroids	MPD 1 mg/kg once followed by taper 0.25 mg/kg^2^, q 6 hours for 21 days for ARDS	MPD 1 mg/kg once followed by DEX 8 mg bid for 6 days for tracheal stenosis	DEX 20 mg once, followed by 10 mg bid for ARDS	MPD 20 mg, q 6 hours followed by taper for a total of 17 days for ARDS	DEX 10 mg once for stridor
Proning	Attempted	No	Attempted	Attempted	Yes
Expired	No	No	Yes	Yes	Yes

Previous reports have shown that 11% of patients who underwent tracheal intubation were noted to have laryngotracheal injuries on endoscopic evaluation six to 12 months after extubation [10]. The length of time to the onset could be variable from one to six months [11,12]. We described two cases of tracheal stenosis, one case of tracheomalacia, and two cases of laryngeal edema, which occurred after mechanical ventilation due to COVID-19.

The risk factors of upper airway complications reported in the literature are prolonged intubation, over-inflation of the tracheal cuff, trauma-induced during intubation, tube displacement after intubation, and endotracheal tube size and the material itself [13,14]. In patients with COVID-19 infection, it is unclear if damage to the larynx and trachea could be caused directly by the COVID-19 virus or if an inflammatory cascade produces laryngeal and tracheal injury. Treatment strategies, including the extensive use of high-dose steroids, may also be a factor. In addition, four of our cases were obese patients, which could be associated with an increased risk of developing post-intubation tracheal stenosis [15]. Maintaining endotracheal cuff pressure at 20-30 cm H_2_O may help to prevent upper airway complications [16]. This would be especially important since many of these patients were moved often during their stay, especially with the use of prone positioning to treat severe COVID-19-associated hypoxemia. In general, the timing of tracheostomy is debatable, and the benefits of early tracheostomy in preventing tracheal injury are unclear. The performance of tracheostomy was unusually delayed (often up to 21 days) in COVID-19 respiratory failure due to risk of viral exposure of healthcare workers during aerosol-generating procedures [8].

A tracheostomy in most studies was considered late if performed after 10-15 days. This difference may have played a role in tracheal injury, and earlier tracheostomy to prevent tracheal stenosis should be investigated further. In a study that examined parameters associated with a need for tracheostomy in COVID-19 patients, the Sequential Organ Failure Assessment (SOFA) score at ICU admission, with a cut-off point of 4.5, was the only variable associated with the need for tracheostomy due to prolonged mechanical ventilation, which could help guide a decision on timing of tracheostomy [17]. Finally, early use of antivirals may decrease viral load and either direct tissue injury or injury from associated inflammation.

Also of interest is that while steroids themselves can decrease inflammation and are used to treat laryngeal edema, the effects of high-dose steroids for long periods, such as those used in COVID-19 may also be damaging to tracheal tissue in vivo, similar to their ability to impair the healing of tracheal anastomoses in a rat model [18]. Although overall beneficial, the optimal duration and dosing of steroids in COVID-19 are still debated.

## Conclusions

Upper airway complications, including tracheal stenosis, tracheomalacia, and laryngeal edema, should be considered in COVID-19 patients with respiratory distress after liberation from mechanical ventilation. The prevalence remains unknown in patients after severe COVID-19 infection. More data is needed to draw more evident conclusions and compare the data with those of COVID-19-negative patients.
